# Effect of 2-Mercapto-1-methylimidazole on the Electrodeposition of Nickel on an Ordered Au(111) Electrode

**DOI:** 10.1021/acsomega.4c00154

**Published:** 2024-04-10

**Authors:** Chiu-Ching Liao, Cheng-Yeh Chang, Shuehlin Yau

**Affiliations:** Department of Chemistry, National Central University Chungli County, Taoyuan 320317, Taiwan, Republic of China

## Abstract

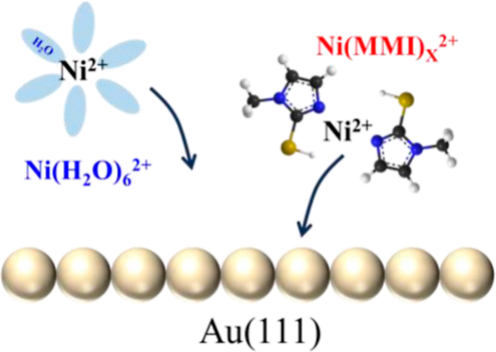

A nickel film electroplated onto a metal substrate can be used as a catalyst for water splitting and a magnetic material for spin valves. Although the nucleation and growth of Ni on Au(111) have already been examined with in situ scanning tunneling microscopy (STM), the current study provides new insights of the structure of the first layer of Ni on an ordered Au(111) electrode in 0.1 M KSO_4_ + 1 mM H_2_SO_4_ + 10 mM NiSO_4_ (pH 3). Prolonged STM scanning of the Ni monolayer on a Au(111) electrode revealed interfacial mixing to produce a surface alloy, initially assuming segregated Ni domains and later transforming them to a homogeneous Ni/Au phase. The formation of the Ni/Au(111) surface alloy affected the structure of the subsequent bulk Ni deposition. The inclusion of 2-mercapto-1-methylimidazole (MMI) in the deposition bath incurred Ni deposition at a less negative potential and a faster rate, resulting in an overall 5.3 times more Ni deposited on the Au electrode in potentiodynamic experiments. MMI molecules were adsorbed on the Ni deposit to prevent Ni dissolution in the Au(111) electrode. MMI could catalyze the presumed rate-determining step from Ni^2+^ to Ni^+^ en route to the metallic Ni. The resultant Ni film with MMI had a 3D texture without a preferred crystal orientation on the Au electrode, as opposed to a layer type growth of Ni on Au(111) without MMI.

## 1 Introduction

Nickel deposition on the reconstructed Au(111) surface has been considered as a model system to study the nucleation and growth of heterogeneous metallization. The use of scanning tunneling microscopy (STM) has enabled a direct view of the preferential nucleation at the elbows of the herringbone pattern and anisotropic growth in the ⟨110⟩ direction of the reconstructed Au(111) surface.^1−4^ The Au(111)-supported Ni thin film has an ordered 2D moiré structure, resulting from the mismatch in lattice constants of the Ni and Au substrate. The anodic dissolution and passivation of Ni(111) and Ni(100) electrodes are also examined with in situ STM.^5−7^

Although the dissolution of the Ni deposit into the Au(111) electrode was inferred from the morphologic STM images,^8^ the atomic structure of this Ni/Au(111) surface has not been reported until now. The Ni/Au interfacial mixing is also found in a vacuum, where single Ni atoms embedded in an Au substrate are imaged with STM. The resulting Ni/Au alloy can be a catalyst for the steam reforming of methane.^9,10^

Electrodeposition of Ni has been conducted in a pH 3 W bath (a mixture of NiCl_2_, NiSO_4_, and boric acid). With a standard reduction potential (−0.257 V vs SHE), Ni^2+^ and H^+^ would be reduced at the same time, leading to hydrogen permeation in the Ni deposit and pH change at the interface.^11,12^ This can be detrimental to the applications of the fabricated Ni film. To remove this interference Ni deposition can be performed in ionic liquids,^13^ producing a noncrystalline Ni film.^14^

Additives, such as coumarin and l-proline, are added to affect the kinetics and thermodynamics of Ni deposition,^15,16^ which enable the control over the grain size, crystallinity, orientation, chemical reactivity, and physical strength of the Ni deposit.^17^ More recently, the importance of using an additive in facilitating Ni superfilling in submicrometer trenches has been illustrated.^16^ Electrodeposition of Ni and Zn alloys in a sulfate bath is reported, producing a corrosion-resistant film on a mild steel.^18^

**Scheme 1:**
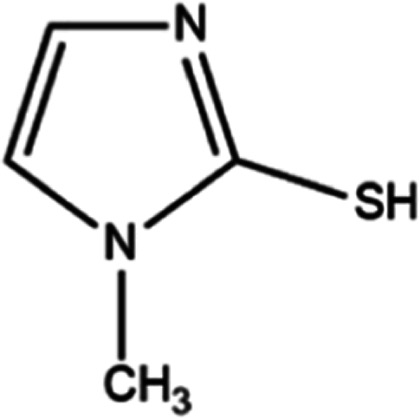
Molecular Structure of MMI

The current study employed in situ STM to study Ni deposition on an ordered Au(111) electrode with and without 2-mercapto-1-methylimidazole (MMI). The structure of the MMI is shown in Scheme 1. The ordered Au(111) electrode was prepared by the annealing-and-quenching method,^19^ but became pitted after MMI’s adsorption.^20^ The structure and kinetics of Ni deposition on the MMI-modified Au(111) electrode were examined.

## 2 Experimental Section

The Au(111) electrode used to conduct the STM or voltammetric experiment was a single-crystal bead made out of a polycrystalline Au wire. The Au electrode was first annealed with a hydrogen torch and then quenched in hydrogen-saturated Millipore water (resistivity 18.2 MΩ·cm). The Au(111) electrode immersed from the quenching tube would be covered with a thin film of water, which prevented the adsorption of contaminants in the ambient. The resultant Au(111) electrode could assume the typical herringbone pattern or the elongated lines, depending on the annealing conditions.

All voltammetric experiments were performed with a three-electrode configuration cell equipped with a Ag/AgCl reference electrode and a Pt counter electrode. The STM cell had two Pt wires acting as the counter and quasi-reference electrodes.^21−23^ The as-prepared Au(111) electrode was confirmed by conducting voltammetric experiments in 0.1 M K_2_SO_4_ + 1 mM H_2_SO_4_ (the base electrolyte), and 10 mM H_3_BO_3_ and 10 mM NiSO_4_ were added in the Ni deposition experiments. All potentials reported here are converted to a Ag/AgCl scale by comparing the voltammograms obtained with Pt and Ag/AgCl as the reference electrodes in the same electrochemical cell. These results are shown in the Supporting Information (Figure S1).

A STM scanner was a typical A-head (Veeco, Santa Barbara, CA) with a maximal scan size of 500 nm. The tip was a tungsten wire etched by AC in 6 M KOH. A film of Apeazon wax was applied to insulate the tip. The potentials of the tip electrode and the feedback current were usually 0.3 V and 1 nA, respectively. All STM images presented in this report were acquired in the constant-current mode.

Sulfuric acid and potassium sulfate were purchased from Showa Chemicals (Tokyo, JP). Nickel(II) sulfate hexahydrate (NiSO_4_·6H_2_O, >99.99%) and 2-mercapto-1-methylimidazole (MMI, > 99%) were purchased from Sigma-Aldrich (St. Louis, MO). Boric acid (H_3_BO_3_, >99.99%) was obtained from Acros Organics (Geel, Belgium). Triple-distilled water (Lotun Technology Co., Taipei) was used to prepare all electrolytes. All chemicals were used as received.

## 3 Results and Discussion

### 3.1 Cyclic Voltammetry

#### 3.1.1 Ni Deposition on Au(111) without MMI

**Figure 1:**
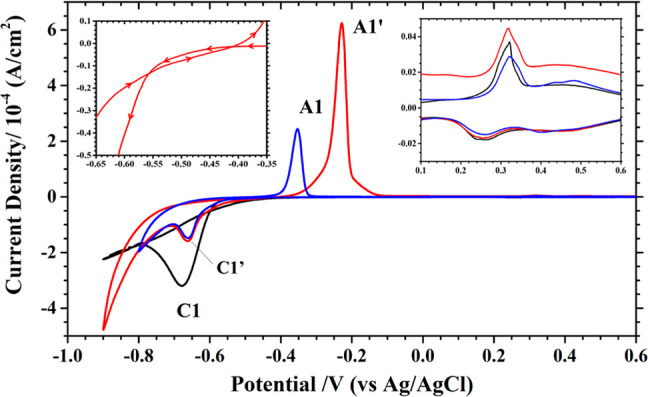
CVs recorded at 10 mV/s from 0.6 to −0.8 V (blue line) and to −0.9 V (red line) with an ordered Au(111) electrode in 0.1 M K_2_SO_4_ + 1 mM H_2_SO_4_ (pH 3) + 10 mM H_3_BO_3_ + 10 mM NiSO_4_. The inset at left and right corners show features associated with the Ni nucleation loop and the Au(111)-( × 22) to (1 × 1) phase transition. The black line was recorded in 0.1 M K_2_SO_4_ + 1 mM H_2_SO_4_.

The as-prepared Au(111) electrode was first characterized with cyclic voltammetry in a pH 3 sulfate solution (0.1 M K_2_SO_4_ + 1 mM H_2_SO_4_) with the potential being cycled at 10 mV/s between 0.6 and −0.9 V. The resultant CV is shown as the black line in Figure 1, featuring a broad peak C1 at −0.68 V and a minor feature at 0.32 V in the negative- and positive-going potential scans. The latter is highlighted by the inset shown at the upper-right side of Figure 1. This is attributed to the adsorption of (bi)sulfate anion and the coupled phase transition from the reconstructed Au(111) to the (1 × 1) phase.^19^

The C1 peak is due to the proton reduction reaction (PRR) at the Au electrode, formulated as 2H^+^ + 2e^–^ → H_2_. Given a standard reduction potential for a PRR of −0.197 V at room temperature, this equilibrium potential is expected to shift to −0.374 V as the pH is raised to 3. However, PRR at the Au electrode is sluggish, resulting in a peak −0.306 V more negative than the equilibrium potential of −0.374 V. The broad peak shape of C1 reflects that PRR is diffusion controlled at the Au electrode in the pH 3 medium.

For the nickel (Ni^2+^) reduction reaction (NRR), an equilibrium potential of −0.514 V is calculated for the 10 mM NiSO_4_ from a standard reduction potential of −0.454 V. The negative potential scan at 10 mV/s from 0.6 V resulted in a reduction peak at −0.66 V (C1′), followed by a sharp increase in current in the pH 3 sulfate solution containing 10 mM NiSO_4_, as shown by the blue and red lines in Figure 1.

The current stayed negative in the positive potential sweep from −0.8 (or −0.9 V) until −0.5 V, leading to an anodic peak at −0.35 (A1) [or −0.23 V (A1′)] with 0.26 or 0.61 mA/cm^2^ peak current.

PRR and NRR occurred simultaneously at the Au electrode in these potentiodynamic experiments, resulting in a smaller, not larger, current density than that of PRR alone, as reflected by the relative size of the C1 and C1′ peaks, as seen in Figure 1. This result is explained by noting that the kinetics of PRR depend on the chemical nature of the electrode. As a comparison, PRR and NRR also occur at the same time on a Pt(111) electrode at −0.51 V,^24^ which yielded a higher current than that of PRR alone. This contrast implies that Ni deposition does not impede the kinetics of PRR at the Pt electrode but suppresses PRR at the Au electrode.

The sharp increase of current at −0.75 V in the negative scan is mostly associated with water reduction (H_2_O + 2e^–^ → H_2_ + 2OH^–^), which is catalyzed by the multilayer Ni deposit on the Au electrode, as the Ni electrode is good at this reaction.^25^ However, neither Ni/Au(111) nor bare Au(111) is capable of oxidizing hydrogen.

The positive potential sweep at 10 mV/s from −0.8 (or −0.9 V) to 0.6 V resulted in an anodic peak A1 at −0.35 V (or A1′ at −0.23 V). They are the counter features of C1′ and ascribed to the stripping of the Ni deposit from the Au electrode. They are both more positive than the calculated equilibrium value of −0.514 V for Ni^2+^ reduction. The thickness of the Ni deposit on this Au electrode is inferred from the integrated charge of A1 or A1′, resulting in 1.5 and 3.0 monolayers of Ni on the Au(111) electrode. [A monolayer of Ni is defined as a Ni(111) plane with a lattice constant of 2.5 Å.] The complete reduction of Ni^2+^ to one monolayer of metallic Ni would consume 600 μC/cm^2^.

It is interesting to see that the Ni deposits obtained by potential sweeping to −0.8 and −0.9 V were stripped off at different potentials (A1 and A1′). As C1′ stayed in the same potential in both scans, it is unlikely that two stripping peaks result from an artifact or faulting of the reference electrode. This result is found in previous studies on Ni deposition on a carbon electrode, which is reconciled with the impregnation of hydrogen in the Ni deposit.^26−28^ The current Ni/Au(111) system can be more complicated by the dissolution of Ni into the Au electrode.^8^

The effect of pH on the stripping of Ni was also considered. Because Ni deposition was coupled with PRR, this would consume H^+^ and raise the pH value at the vicinity of the Au electrode.^12^ One would expect to see a greater rise in pH when the potential was swept to −0.9 V than to −0.8 V. We conducted voltammetric experiments on Ni deposition and stripping in pH 4 and 5 sulfate media. As the pH of the electrolyte increased from 3 to 4 and 5, Ni deposition and stripping peaks were both negatively shifted (Figure S2), which is opposite to the results, as shown in Figure 1. Therefore, the Ni stripping peak did not result from a change in pH at the interface of the Au electrode.

The current efficiency (η) of Ni deposition in the voltammetric experiment is calculated by dividing the Ni stripping charge (Q_NRR_) by the overall charge in the negative-going potential sweep (Q_red_). This analysis yields η values of 34 and 26% when the potential was swept to −0.9 and −0.8 V. The remaining portions (66 and 74%) are attributed to the PRR and water reduction reaction in the pH 3 sulfate +10 mM NiSO_4_.

A detailed portrait of the onset potential for Ni deposition in the first cycle is shown at the top left of Figure 1. A hysteresis or a loop in the negative and positive sweeps is noted, which is also found with a carbon electrode.^26^ This hysteresis was observed only in the first scan, not in the following scans, as shown in the Supporting Information (Figure S3). This feature signals a nucleation overpotential or a shift of the equilibrium potential for Ni^2+^ reduction on the Au(111) electrode. This variation in the CV profiles could be traced to changes in the structure of the Au(111) electrode, as revealed by the STM results shown in Figure S4. The elongated lines of a well-ordered reconstructed Au(111) surface were chopped into short segments (<200 Å) after it was subjected to Ni deposition and stripping. It is assumed that an ordered Au(111) surface was more stable against Ni nucleation, and an increase in overpotential resulted.

This change in the surface structure of the Au(111) electrode is likely responsible for the variation in the peak shape, associated with the reconstruction to (1 × 1) phase transition at 0.32 V. This feature highlighted in the inset at the upper right of Figure 1 is seen with not only the bare but also the Ni-plated Au(111) electrode. It seems that the Au(111)-(1 × 1) structure induced by Ni deposition rapidly reverted to the reconstructed phase (<1 min) after the Ni deposit was removed. This phase transition was observed with the STM (Figure S4).

#### 3.1.2 Ni Deposition on the MMI-Modified Au(111)

The effect of adsorbed and solution MMI on the Ni deposition on the Au(111) electrode were examined in the pH 3 sulfate media, with the aim of deconvoluting the surface and diffusion processes pertaining to Ni^2+^ reduction. Three Au(111) samples with different MMI coverages were prepared by immersing in 0.01, 0.1, and 1 mM MMI dosing solutions for 30 s. They were first examined with voltammetry in a pH 3 sulfate media. The obtained CVs (Figure S5) show a pair of redox peaks at −0.2 V, which is ascribed to the protonation and restructuring of the MMI adlayer.^20^ Different current densities were seen with these samples, suggesting different MMI’s coverages. The current density and MMI coverage on the Au electrode increase with [MMI].

**Figure 2:**
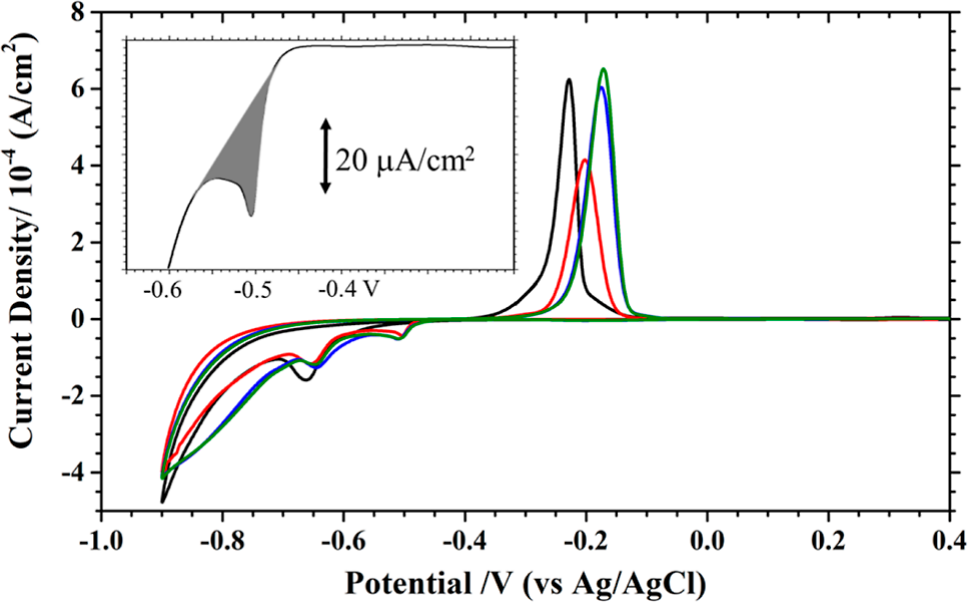
CVs recorded at 10 mV/s with Au(111) electrodes without (black line) and with MMI modification in pH 3 sulfate +10 mM NiSO_4_. The red, blue, and green lines were recorded with Au(111) electrodes premodified with 0.01, 0.1, and 1 mM MMI. The inset highlights the first negative potential scan obtained with 0.01 mM MMI-coated Au(111), resulting in a prepeak at −0.5 V, whose charge is integrated as the shaded area.

The Ni deposition on the MMI-modified Au(111) electrode was then examined in a pH 3 sulfate solution containing 10 mM NiSO_4_. The potential was cycled at 10 mV/s between 0.4 and −0.9 V, yielding CVs, as shown in Figure 2. (The red, blue, and green lines correspond to [MMI] of 0.01, 0.1, and 1 mM.) A minor peak at −0.51 V, prior to the main reduction peak at −0.66 V, was observed for all MMI-modified Au electrodes, as highlighted in the inset of Figure 2. This feature was not seen with the bare Au electrode (black line in Figure 2).

This prepeak at −0.51 V was also seen with the Au electrode dosed with 0.1 mM MMI (Figure S6). The charge contained in this peak increases from 110 to 130 ± 5 μC/cm^2^ with a higher [MMI]. The peak potential of −0.51 V did not change with the MMI coverage on the Au electrode. This prepeak was seen only with the presence of Ni^2+^ and MMI, which implies that the MMI admolecules could shift the reduction of Ni^2+^ to a less negative potential. This result resembles the renowned underpotential deposition (UPD).

As the potential sweep was reverted at −0.9 V, the current stayed negative until −0.4 V, where the current became positive, leading to a peak at −0.17 V, which is ascribed to the stripping of the Ni deposit. As compared with a bare Au(111) electrode, this Ni stripping peak shifts positively by 60 mV. As seen with the STM (described below), this result is explained by the segregation of MMI admolecules from the Au electrode onto the Ni deposit. This MMI-capped Ni deposit became more difficult to strip off from the Au electrode.

The amounts of Ni deposit on these MMI-coated Au(111) electrodes are found to be 4.1, 5.8, and 6.0 ML with increasing dosing [MMI]. Compared with the 5.0 ML of Ni on the unmodified Au(111), this MMI modification promoted Ni deposition, except with the lowest dosage of 0.01 mM MMI. This difference suggests that the MMI coverage affected the kinetics of Ni deposition on the Au electrode.

These CV profiles were not stable against potential cycling as the Ni stripping peak increased with scanning. The maximal Ni thickness reached 4.9, 8.7, and 9.4 ML at the 10th potential cycles, as shown in Figure S7. The current efficiencies for Ni deposition are found to be 37, to 45 and 47% with increasing [MMI], up from 34% found with no MMI modification. This promotion effect of MMI on Ni deposition resembles that of sulfamate, which is presumed to be coadsorbed with the Ni adatom on the Au electrode.^15^

#### 3.1.3 Ni Deposition on Au(111) with Solution MMI

**Figure 3:**
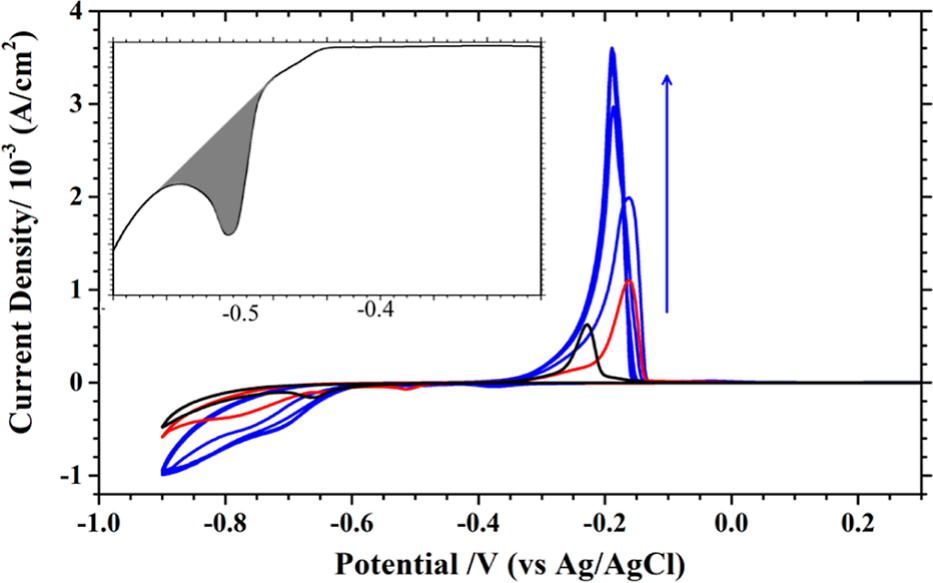
CVs recorded at 10 mV/s with Au(111) in pH 3 sulfate +10 mM NiSO_4_ solution without (black line) and with (red, the first cycle and blue lines, 2–6 cycles) 0.01 mM MMI in the deposition bath. The inset shows the first negative scan from −0.28 and −0.6 V and the charge integration.

The effect of solution MMI on Ni deposition on Au(111) is now described. Shown in Figure 3 are the first (red) and second to sixth (blue) potential cycles at 10 mV/s in the presence of 0.01 mM MMI dissolved in pH 3 sulfate +10 mM NiSO_4_. These profiles have characteristics the same as those of the MMI-modified Au(111) electrode (Figure 2) but with a notably higher current density.

The first negative-going sweep from −0.2 to −0.6 V is shown in the inset of Figure 3 to highlight the Ni deposition in the presence of MMI. The reduction current commenced at −0.44 V and increased to a maximum at −0.51 V. This marks the initiation of Ni deposition on the Au electrode. Its charge is found to be 160 ± 10 μC/cm^2^, compared with 110 μC/cm^2^ observed with the MMI-modified Au electrode. If a pseudomorphic Ni adlayer on the Au(111) electrode is assumed, the charge for fully discharged Ni should be 420 μC/cm^2^. The 260 μC/cm^2^ (420–160) deficit can be due to the sluggishness of the process and incompleteness of the Ni adlayer in the prepeak.

The CV results indicate that the solution MMI exerted a marked effect on Ni deposition on the Au(111) electrode. This might be at odds with the fact that the MMI admolecules are desorbed from the Au electrode at *E* < −0.25 V, but this process was slow and incomplete at −0.51 V in this potentiodynamic experiment. The Au(111) electrode was mostly pitted and minorly reconstructed, as seen with the STM (Figure S8).^20^ Moreover, the presence of Ni^2+^ could change the electrode process, as Ni^2+^ could interact with MMI molecules.

In the positive going sweep from −0.9 to 0.15 V, the oxidation current commenced at −0.4 V, leading to a Ni stripping peak at −0.17 V, as seen with the MMI-modified Au electrode (Figure 2). The positive end of this Ni stripping peak is highlighted in Figure S8, showing a small peak at −0.12 V, which is ascribed to the stripping of the first Ni layer.

The CV profile was not stable against potential cycling between −0.9 and 0.2 V. The Ni stripping peak grew notably and shifted slightly negatively with potential cycles. The peak current of the second cycle is nearly double (1.8 times) that of the first sweep, and the margin of increase decayed in the following cycles. The peak current reached the maximal value in the eighth cycle, producing ∼27 ML Ni on the Au electrode, representing a 5.3 times increase from that (5 ML) with no MMI.

**Figure 4:**
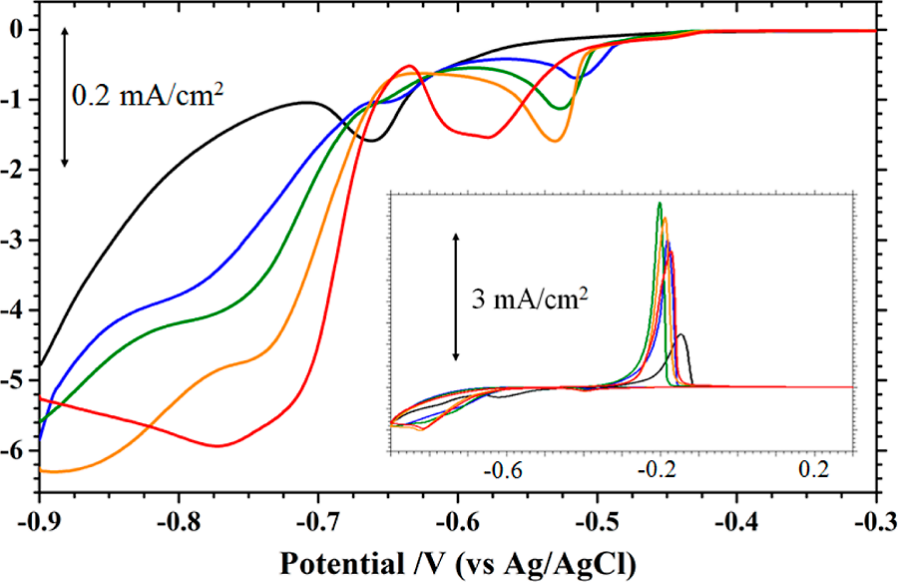
CVs recorded at 10 mV/s with the Au(111) electrode in pH3 sulfate solution +10 mM NiSO_4_ without (black line) and with 10, 25, 50, and 100 μM MMI (blue, green, orange, and red lines). The inset reveals the potential cycles gave rise to the highest peak currents of Ni stripping in these solutions.

The effect of [MMI] on Ni deposition was substantiated by conducting voltammetry with 10, 25, 50, and 100 μM MMI in the deposition bath. The first negative potential scans in these media are shown in different colors in Figure 4. A prepeak was noted in each trace with the peak current increasing and potential shifting negative as [MMI] increased. With 0.050 and 0.100 mM MMI, the prepeaks were broad and centered at −0.53 and −0.6 V, which are more negative than an equilibrium potential of −0.514 V. An amount of 880 ± 10 μC/cm^2^ charge was contained in these peaks, which is enough to have a bilayer pseudomorphic Ni on Au(111).

The subsequent positive scan resulted in a Ni stripping peak at −0.2 V, which increased with the number of potential cycles. The cycles giving rise to the highest stripping peaks with different [MMI] are shown in the inset of Figure 4. The amounts of Ni deposit evaluated from the stripping peaks are summarized in Table 1. The current efficiencies for Ni deposition are statistically equivalent (lying between 68 and 72%) with 10 to 100 μM [MMI]. The aforementioned hysteresis loop (shown in the inset of Figure 1) is not observed with MMI.

**Table 1:** Deposition, Stripping Charges, and Current Efficiency for the Cycle Yielding the Maximal Thickness of the Ni Deposit on the Au Electrode

MMI conc. (μM)	deposition (mC/cm^2^)	stripping (mC/cm^2^/ML)	current efficiency (%)
0	9.17 ± 0.2	3.0 ± 0.1/5.0	33.1 ± 0.9
10	23.5 ± 0.5	16.0 ± 1.0/26.7	68.2 ± 3.4
25	24.4 ± 0.1	17.0 ± 1.3/28.3	69.7 ± 5.6
50	23.1 ± 2.2	16.6 ± 2.0/27.6	71.6 ± 2.3
100	25.6 ± 2.7	18.0 ± 1.9/30.0	69.6 ± 4.8

The effect of organic additives in metal electrodeposition can vary with the chemical nature of metals because of different reduction potentials. For example, the standard reduction potential of Cu^2+^ is 0.6 V more positive than that of Ni^2+^. Thiol molecules are strongly adsorbed on the Au electrode at a potential where Cu deposition commences. They could thus block Cu nucleation and impede Cu UPD.^29,30^ By contrast, Ni deposition occurs at a much more negative potential, where the thiol molecules (such as MMI) interacted weakly with the Au substrate. Because thiol molecules are strongly adsorbed on the Ni metal,^31−33^ they can segregate on the Ni deposit and stabilize the Ni/Au(111) electrode.

**Scheme 2:**
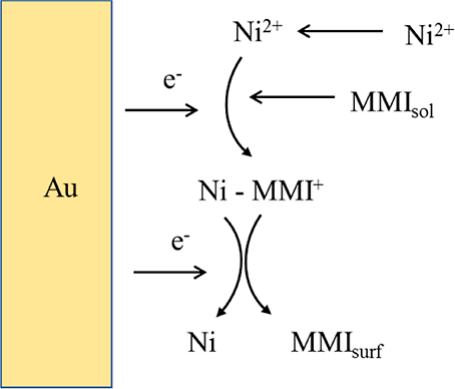
MMI-Steered Ni^2+^ Reduction Mechanism at an Au Electrode

The obtained CV results suggest that MMI catalyzed the NRR on the Au(111) electrode. The reduction of Ni^2+^ can proceed in two one-electron steps, involving Ni^+^ as the intermediate species.^34^ If the first reduction reaction Ni^2+^ + e^–^ → Ni^+^ is rate determining, then MMI facilitated this reaction by forming a Ni-MMI^+^ complex. The precursors for the Ni deposit, including Ni^2+^ and Ni(MMI)^+^, accumulated at the Au interface with potential cycling, leading to an increase in the amount of Ni in the first 10 cycles. This proposed reaction scheme is shown in Scheme 2, where the interfacial MMI could come from the diffusion of solution MMI_sol_ and the release from the second reduction step MMI_surf_. [MMI] at the interface of the Au electrode gradually reaches a steady state with potential cycles.

### 3.2 In Situ STM

The current STM study explored a number of unaddressed issues on Ni deposition on Au(111).^2,3^ In addition to the morphologic changes of Au(111) due to Ni dissolution,^8^ in situ STM atomic images were obtained to reveal the spatial structure of the Ni/Au(111)-alloyed surface. The adsorption configuration and spatial structure of MMI adsorbed on Au(111) are already reported.^20^ The effect of MMI on Ni plating on the Au electrode was also examined with STM here.

#### 3.2.1 Ni Deposition on Au(111) without MMI

**Figure 5:**
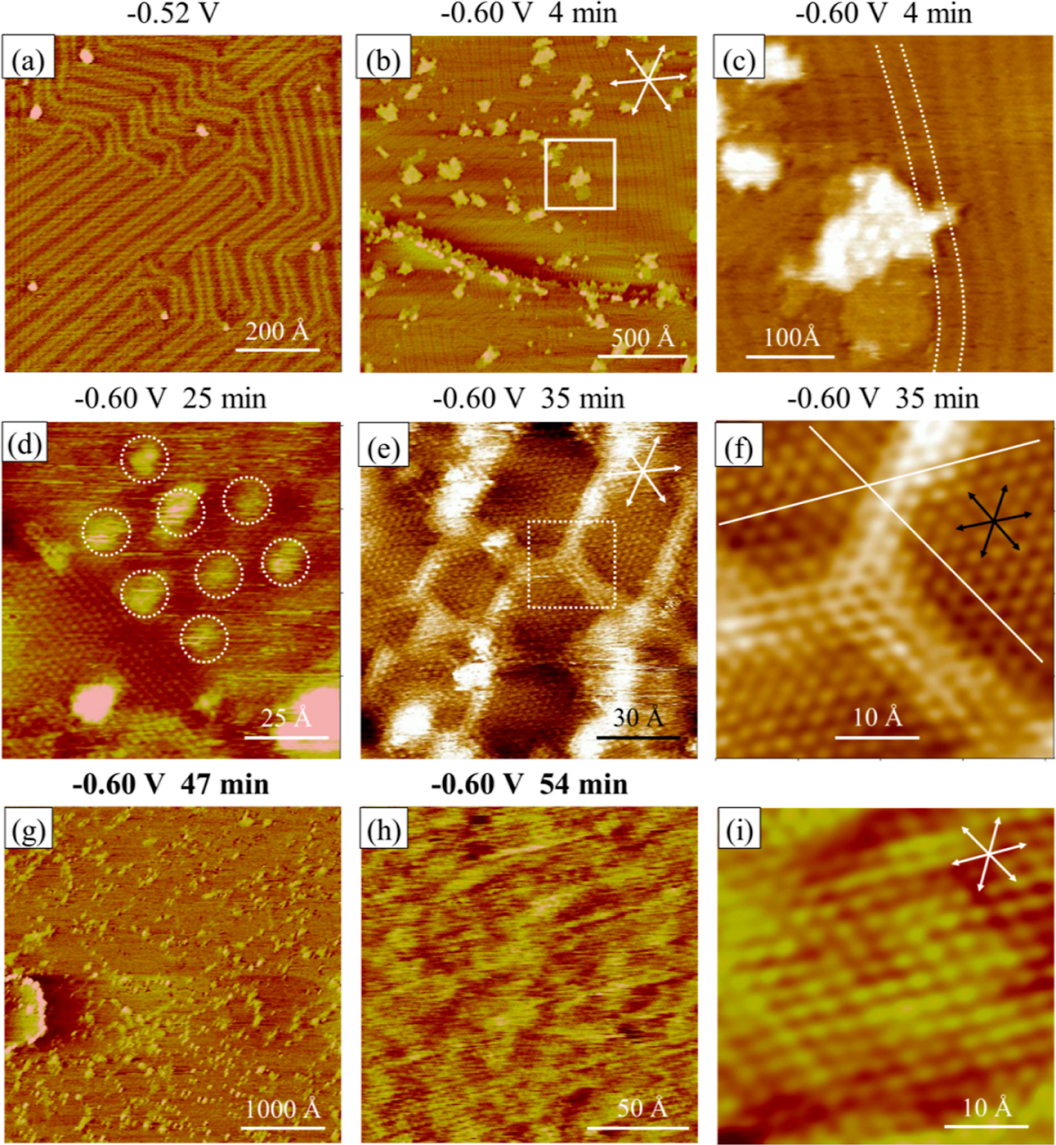
STM images showing the nucleation (a) and growth (b-g) of Ni on a bare Au(111) electrode in pH 3 sulfate +10 mM NiSO_4_, as the potential was lowered from −0.52 to −0.60 V. Dotted lines marked in (c) indicate distortion in the reconstructed lines near the Ni deposit. Dotted circles in (d) highlight the moiré pattern due to the Ni deposit. Solid lines show the misalignment of atomic rows in two neighboring domains, and dotted lines show a domain wall between two hexagonal arrays (f). The surface structure of a homogeneously mixed Ni/Au(111) is revealed by panels (g–i). These STM images were collected during the same experiment.

The initial stage of Ni deposition on the bare Au(111) electrode is described first. Figure 5a shows a STM snapshot of Au(111) electrode recorded at −0.52 V in pH 3 sulfate media +10 mM NiSO_4_, featuring an elongated (>1000 Å) linear pattern and some herringbone features preferentially aligned in the ⟨121⟩ directions of the Au(111) surface. The 2D periodicities of these structures are 64 Å, indicating the reconstructed Au(111)-( × 22) surface. Pits and protruded dots are noted at the elbows of the herringbone patterns, which are ascribed to the Ni deposit.^2^

This surface state was mostly stable until the potential was shifted from −0.52 to −0.6 V, where protruding 2D islands emerged, as seen in Figure 5b. They are typically 1.8 Å higher than the reconstructed Au(111) surface. The finer STM image shown in Figure 5c reveals structures near these islands, which were bilayer with a Moiré pattern atop a featureless lower layer. These STM images reveal the nucleation and growth processes of Ni on a bare Au(111) electrode, as reported by others.^3,35^ Moreover, the elongated lines in the vicinity of the Ni islands are clearly bent, as outlined by the dotted lines in Figure 5c. This feature can reflect the dissolution of Ni into the Au surface to be substantiated below.

The atomic resolution STM image shown in Figure 5d reveals the atomic structures on a protruding Ni island (top right) and the neighboring Au substrate (lower left). The former features a long-range (∼22 Å) intensity modulation or Moiré pattern, as seen with the multilayer Ni deposit on Au(111) and Pt(111).^3,24^ The latter is a hexagonal array with atomic features aligned in the ⟨110⟩ direction of the Au(111) surface and the nearest neighbor spacing is 2.9 ± 0.1 Å.

After scanning at −0.6 V for 35 min, the ongoing Ni deposition resulted in patchy atomic arrays, as seen in Figure 5e. A portion of this image was filtered with the 2D FT technique to remove noise signals with spatial spacing less than 2.5 Å. The resultant image is shown in Figure 5f, featuring hexagonal arrays segregated by domain walls 7–10 Å thick or 3–5 atomic rows separated by 2.5 ± 0.1 Å.

These segregated hexagonal patches are certainly not expected for an ideally truncated and chemically pure Au(111) surface. Thus, we attribute this unique surface state to Ni–Au(111) interfacial mixing. The corrugated domain walls (Δ*z* = 0.14 Å) are the segregated Ni adatoms in the Au(111) surface. They caused strain in the uppermost layer of the Au(111) surface, forcing some Au atoms to shift from the FCC to HCP sites. This is responsible for the misalignment of atomic features highlighted by two straight lines, as outlined in Figure 5f.

The sample was continuously scanned for 2 h at −0.6 V. The morphology and atomic structure of the sample evolved with imaging. The Ni moiré pattern largely diminished and protruded islands (Δ*z* = 2.2 Å) emerged randomly on the terraces (Figure 5g). High-resolution STM scans were obtained at this stage, revealing distorted hexagonal lattices with the nearest neighbor spacing of 2.9 ± 0.1 Å (Figure 5h,i). Corrugated atomic features (Δ*z* = 0.18 Å) are seen, which are attributed to a homogeneously mixed Ni and Au atoms on the surface.

**Figure 6:**
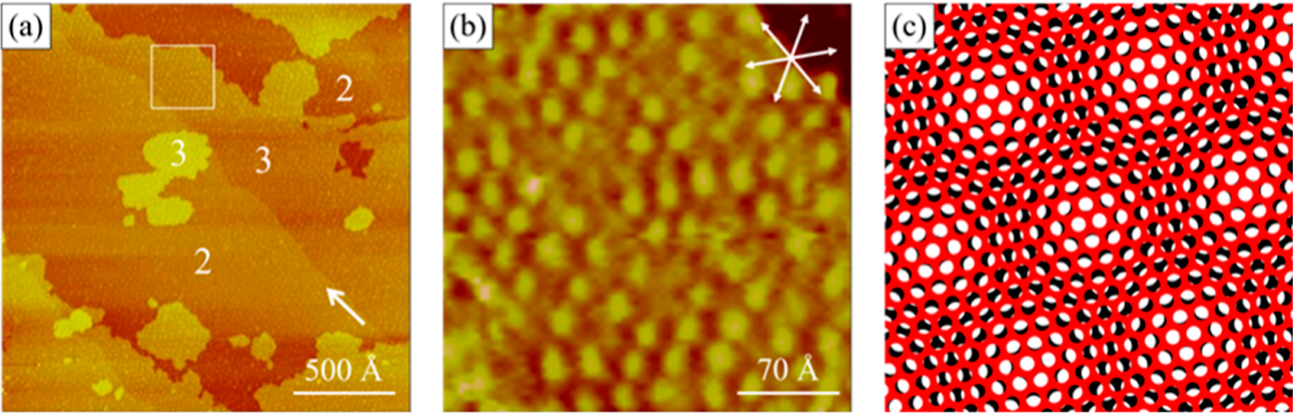
In situ STM images showing the multilayered Ni deposit on the Au(111) electrode at −0.62 V (60 min) in pH 3 sulfate solution +10 mM NiSO_4_. The numbers in (a) mark the layers of the Ni deposit. (b) Zoom-in of (a), showing local moiré patterns in the second layer of Ni. The arrow marked in (a) indicates a step edge in the Au substrate. (c) Ball model for this moiré pattern.

Shifting the potential from −0.60 to −0.62 V resulted in more Ni deposition, giving rise to layered islands on the Au(111) electrode (Figure 6a). The height of these islands is 2.0 Å, suggesting that they were one Ni atom thick. The finer resolution STM scan (Figure 6b) collected on the top of an island reveals local moiré patterns with intensity undulating in the ⟨110⟩ direction of the Au(111) substrate in a periodicity of 22 Å. These results are consistent with multilayer Ni deposit on the Au(111) electrode^2,3^ and is reconciled with the ball model, as depicted in Figure 6c.

Although bulk Ni and Au are immiscible, Au adatoms can dissolve into Ni(110) and Ni(111).^36,37^ The Au adatom is imaged as a local depression in the Ni plane. The STM corrugation heights of chemically different atoms can vary with the imaging conditions and the chemical composition of the tip. Ni deposit is found to dissolve in Ag(111) also, which preferentially occurs at step defects.^38^

Electrodeposition can be used to generate not only interfacial alloys such as Ni on Au(111) but also alloyed coatings to impart specific properties and change the appearance of an object. One modern aspect of electrodeposition involves the square pulse current sequence procedure^39^ to fabricate a number of compositionally modulated multilayered materials, such as ZnFe,^40^ ZnCo,^41^ and ZnNi,^18,42^ on mild steel and others. These alloyed nanocoatings have unique microstructures and electronic properties, eventually leading to superior corrosion resistance. Although new compound materials have been produced, the atomic structure at the electrified interface can be elusive. As illustrated with the current study of Ni deposition on Au(111), in situ STM can be used to probe the atomic structures of these artificial films in the deposition and oxidation stages.

#### 3.2.2 Ni Deposition on Au(111) with Solution MMI

**Figure 7:**
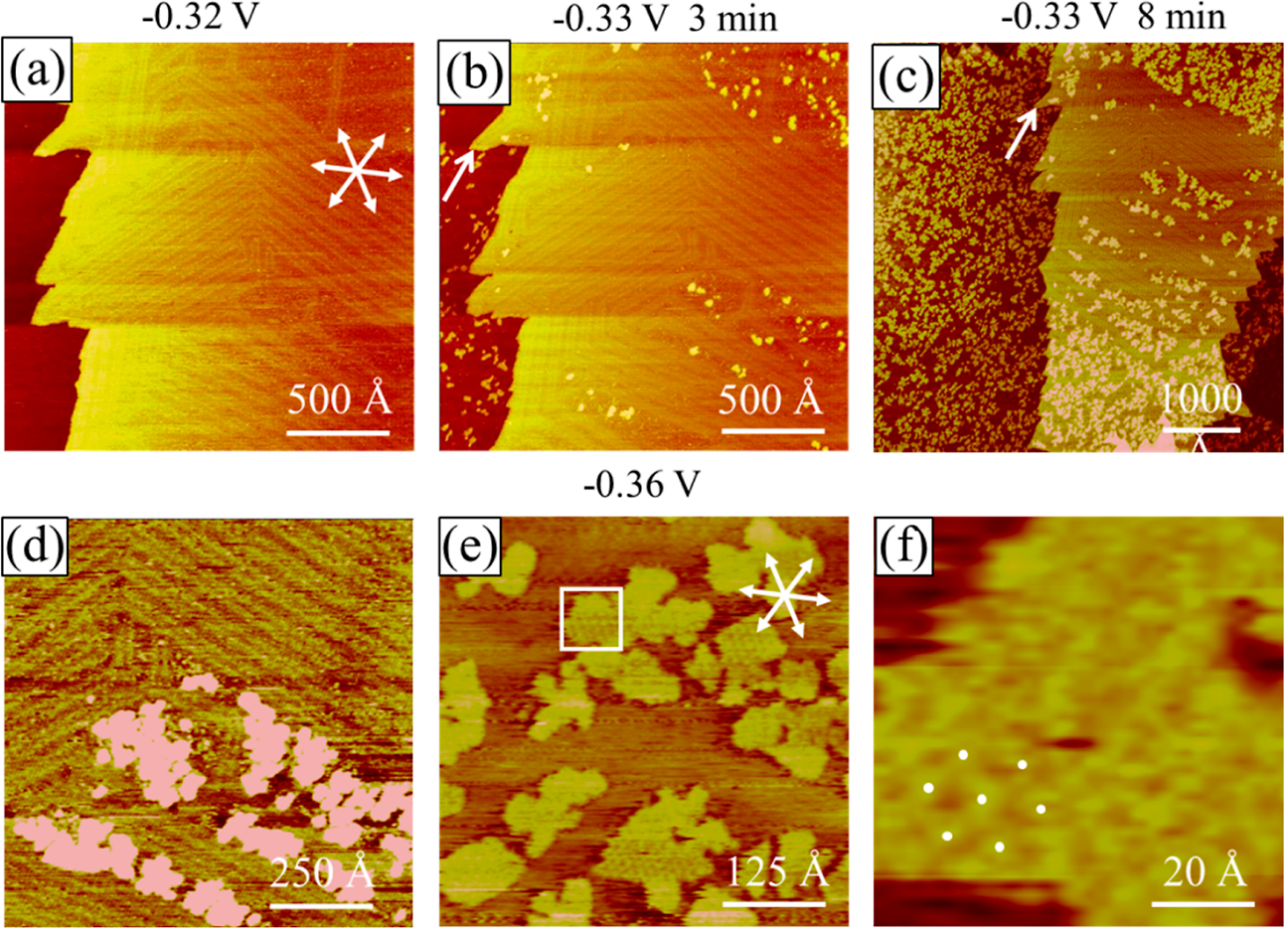
In situ STM images collected with the Au(111) electrode at −0.32 (a), −0.33 (b–d), and −0.36 V (e,f) in pH 3 sulfate +0.01 mM MMI + 1 mM NiSO_4_. Arrows in parts (b,c) mark the same kink site. (d) Highlights the Ni islands and neighboring straight reconstructed lines. (e,f) Hexagonal array on the protruded islands. All STM images were acquired during the same experiment.

The adsorption of MMI on the Au(111) electrode was examined previously,^20^ revealing that MMI molecules form an ordered ( × )R10.9° at 0 V in pH 3 sulfate + 10 mM NiSO_4_ + 0.01 mM MMI. MMI admolecules started to desorb slowly from the Au electrode at *E* < −0.25 V. Holding the potential at −0.32 V for ∼30 min could restore 90% of the reconstructed Au(111) surface, as seen in Figure 7a. The remaining Au(111)-(1 × 1) domains could be occupied by MMI admolecules, probably adsorbed in a disarray, according to a high-resolution STM scan (not shown).

The potential was then shifted negatively from −0.32 to −0.33 V to deposit Ni, which yielded protruded dots preferentially on the (1 × 1) domains, as seen in Figure 7b, collected 3 min after scanning at −0.33 V. Dots are 20–30 Å wide and 1.8 ± 0.1 Å high (with respect to the reconstructed pattern). If the electrolyte was free of NiSO_4_, only the reconstructed Au(111) surface was seen, as shown in Figure S8. It is possible that the MMI additive induced a positive shift of potential for Ni deposition on the Au electrode, as reported with sulfamate.^15^

Seeing Ni deposition on the Au(111) electrode at −0.33 V seems to be at odds with the CV result (Figure 3), which shows no current at −0.33 V. This inconsistency is explained by the kinetics of Ni deposition and the time scales for the CV and STM measurements. The large overpotential for Ni deposition seen with CV reflects that this is a slow process and could be overlooked with voltammetry when the potential was swept at 10 mV/s. On the other hand, STM imaging is slow and usually requires minutes to hours to have a resolution good enough to show changes at the electrode. STM imaging of Ni deposition was thus performed at some fixed potentials.

Protracted scanning at −0.33 V for 8 min resulted in growing dots distributed uniformly on the 4000 × 4000 Å scan shown in Figure 7c, except for one area on the terrace on the right-hand side of Figure 7c. This particular area was scanned earlier (Figure 7a,b), which could be blocked by the STM tip partially blocking Ni deposition when scanning with +0.35 V bias voltage and 1 nA feedback current. The seesaw-like step in these images (as indicated by the arrows) is used to guard against thermal drift in STM scanning.

These dots expanded laterally and eventually coalesced with their neighbors to form 2D islands, as seen in Figure 7d collected after 10 min scanning at −0.33 V. Moreover, most islands were found to reside on the areas between two paired lines or the FCC domains of the Au(111) surface. The reconstructed linear patterns sitting next to islands are straight as opposed to the bent lines seen without MMI (Figure 5c).

The resultant islands have rugged edges but smooth top surfaces, as revealed by the finer resolution STM scans shown in Figure 7e,f. The limited width (<200 Å) of the islands made it difficult to obtain good-quality STM images. A short-range hexagonal array is seen on a protruding island (Figure 7f). This structure was observed only in the presence of the MMI additive and contrasts markedly with the distorted hexagonal patterns seen without MMI (Figure 5i). This array has a nearest neighbor spacing of 12 ± 0.5 Å with the spot rows 10° off the ⟨110⟩ axis of the Au(111) substrate.

**Figure 8:**
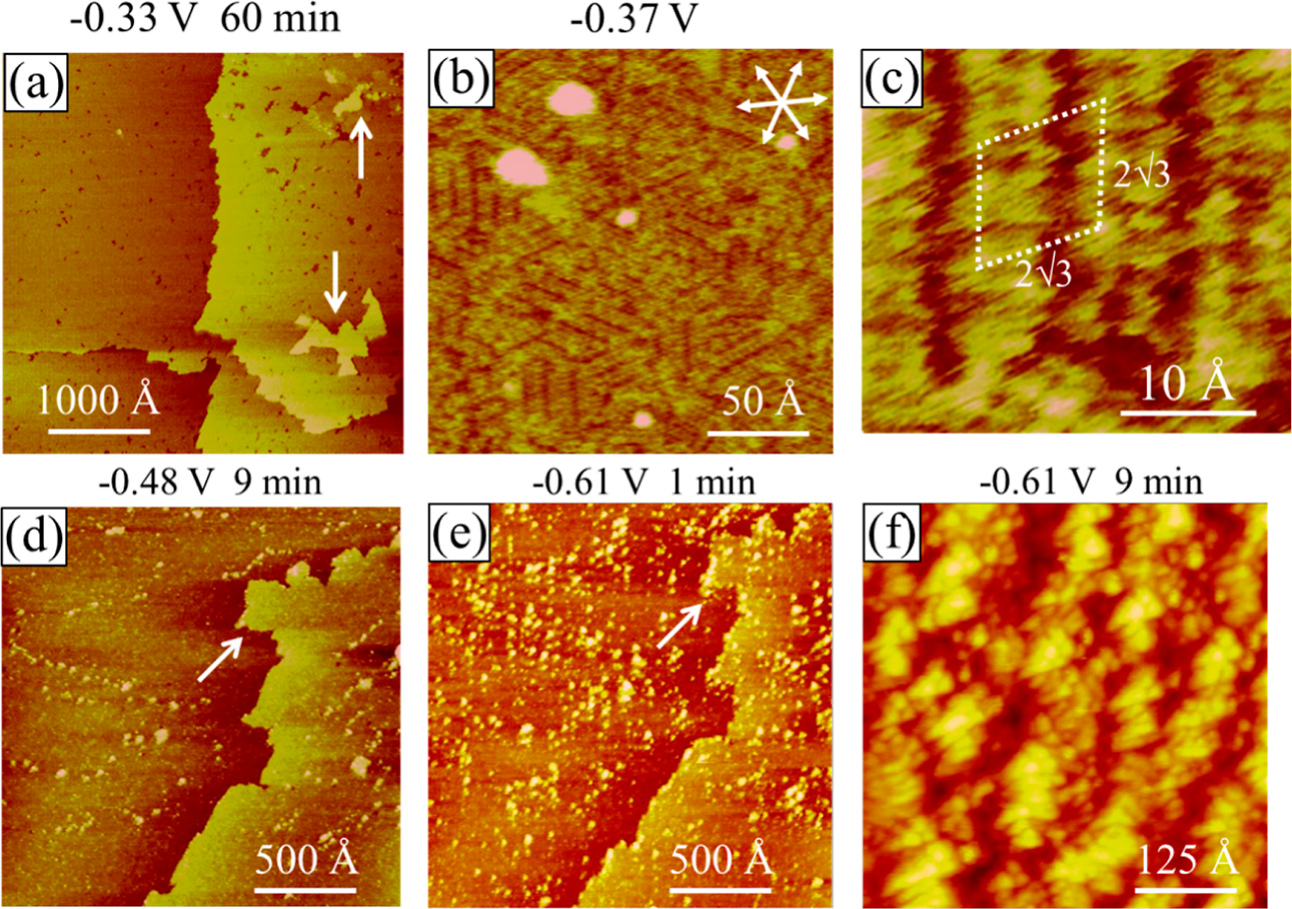
In situ STM images showing the Au(111) electrode nearly (95%) covered with a Ni monolayer at −0.37 V in pH 3 sulfate +0.01 mM MMI + 1 mM NiSO_4_. Protruded islands pointed by arrows (a) are aggregates of Au atoms. Adsorbed MMI spatial structures are seen in (b,c). More Ni deposition occurred at −0.48 V (d) and −0.6 V (e–f).

Holding the potential at −0.33 V for about 1 h yielded a smooth Ni monolayer (Δ*z* = 2.0 Å) with a notable number of pits, as seen in Figure 8a. These pits in the Ni deposit were not filled with prolonged scanning. Meanwhile, well-defined islands (indicated by the arrows in Figure 8a) are found on the smooth terrace. Because no bulk Ni deposition is expected at this potential, they are attributed to aggregated Au atoms, liberated from the Ni-induced phase transition from the reconstructed Au(111) to the (1 × 1) phase.

Finer resolution STM scans shown in Figure 8b and c reveal patchy linear features mostly aligned in the ⟨121⟩ direction of the Au(111) electrode. Paired lines ∼3 Å in width are seen in each domain, which is ascribed to the −N and −S ends of MMI molecules oriented upward on the Au electrode. The two nearest MMI molecules within a row are 3.5 Å apart and two neighboring MMI rows are separated by 10.0 ± 0.2 Å. These results lead to a (2  × 2 )R30°-Ni + MMI adlattice. Because there were three equivalent ⟨121⟩ directions on the Au(111) electrode, MMI molecules could nucleated and grew into three rotational domains, resulting in a patchy appearance of the Ni + MMI adlayer. On average, each domain spans less than 200 Å. This surface structure was stable for at least 30 min, which contrasts with the changing structures seen without MMI (Figure 5d–i). This implies that MMI adsorption stabilized the Ni deposit on the Au electrode and prevented its dissolution into the Au electrode.

Ni bulk deposition was not observed until −0.48 V, where nanometer-sized clusters appeared preferentially at defects (pits) in the first Ni layer, as revealed by Figure 8d, collected 9 min after scanning at −0.48 V. Bulk Ni deposition hastened after the potential was changed from −0.48 to −0.61 V, producing larger Ni clusters within 1 min (Figure 8e) and a thicker film within 10 min (Figure 8f). The rolling hill morphology of the electrode seen in Figure 8f reflects a nodule-shaped Ni deposit. The corrugation height on average is 8 Å high or 4 layers of Ni. The lack of a well-defined shape of the Ni nodule suggests a polycrystalline film.

As Ni metal is readily oxidized in air, it is difficult to prepare ordered SAMs of thiol molecules on a Ni substrate using the traditional dipping method. The current STM study illustrates another way to prepare SAMs of thiol molecules on Ni by electrodeposition on Au or other electrode. If the interested thiol molecules are insoluble in water, ionic liquid can be used as the solvent for conducting Ni electrodeposition.^14^

There are only a few reports on thiol adsorption on the Ni metal. In a vacuum, aromatic thiol molecules adsorbed on the Ni substrate convert to thiolate at 200 K and decompose to S adatoms and organic fragments at room temperature.^32^ Because the (2  × 2 )R30° structure seen in Figure 8c differs from Ni(111)-(2 × 5 )-S,^43^ we presume that the MMI molecule was adsorbed intact on the Ni deposit in the solution. l-cysteine can be adsorbed on the Ni electrode from a mixed water + ethanol solution.^44^

## 4 Conclusions

Cyclic voltammetry and in situ STM have been used to characterize the nucleation, growth, and bulk deposition of Ni on an ordered Au(111) electrode in pH 3 sulfate + 10 mM NiSO_4_ solution with and without MMI as an additive in the deposition formula. The negative-going potential excursion at 10 mV/s from 0.6 to −0.8 V (or −0.9 V) triggered concurrent reduction of H^+^ and Ni^2+^ at −0.5 V, resulting in a low current efficiency for Ni deposition (η_Ni_) of 26 (or 34%). However, the presence of micro molar MMI elevated η_Ni_ to 68% and generated five times more Ni deposit on the Au electrode under the same experimental condition, suggesting that MMI catalyzed the reduction of Ni^2+^. This promotion effect of MMI is also manifested in the positive shift of the potential for Ni deposition. The Ni deposits obtained with negative-going potential sweeps to −0.8 and −0.9 V in the pH 3 sulfate solution were stripped off at −0.35 to −0.23 V, respectively. This positive shift of the Ni stripping potential was observed in the presence of MMI. This correlates with the molecular resolution STM results showing the adsorption of MMI on the Ni deposit. MMI admolecule also inhibited the dissolution of Ni adatoms into the Au(111) substrate, which was observed without MMI. The interfacial mixing between Ni adatoms and Au(111) substrate was characterized by atomic resolution STM imaging, showing that Ni adatoms initially segregated as domain walls separate arrays of Au atoms occupying FCC and HCP domains and transformed into a homogeneously mixed Ni/Au(111) state in 2 h. Ni deposition on the Au(111) electrode proceeded in quasi-layered and three-dimensional manners without and with MMI in the deposition bath, producing smooth and rough Ni coatings on the Au(111) electrode.

## Supporting Information Available

CVs obtained with Au(111) using Pt and Ag/AgCl as the reference electrode; first CV cycle recorded with Ni deposition on Au(111); CVs showing Ni deposition and stripping in pH 3, 4, 5 sulfate media; STM images showing changes in the surface structure of Au(111) electrode before and after Ni deposition; CVs recorded with MMI-modified Au(111); first negative sweep for Ni deposition on Au(111); CVs giving rise to the highest stripping peak of Ni; stripping CV of the first layer of Ni; STM images showing changes of Au(111) electrode during the desorption of MMI admolecule; and STM images showing the moire pattern seen with the multilayer Ni on Au(111) (PDF)
