# Supplementary material for: Effect of 2-Mercapto-1-methylimidazole on the Electrodeposition of Nickel on an Ordered Au(111) Electrode
Source: ACS Omega. 2024 Apr 10;9(16):18304–13. doi: 10.1021/acsomega.4c00154 (PMC11044226; doi:10.1021/acsomega.4c00154)
Supplement: Supplementary file 1 — ao4c00154_si_001.pdf [file ao4c00154_si_001.pdf]

Supporting information for

The Effect of 2-Mercapto-1-methylimidazole on the Electrodeposition of Nickel on an Ordered Au(111) Electrode

Chiu-Ching Liao, Cheng-Yeh Chang, and Shuehlin Yau*

Department of Chemistry, National Central University

Chungli County, Taoyuan City, Taiwan, ROC

Corresponding author: Shuehlin Yau, yau6017@ncu.edu.tw

Submitted to ACS Omega

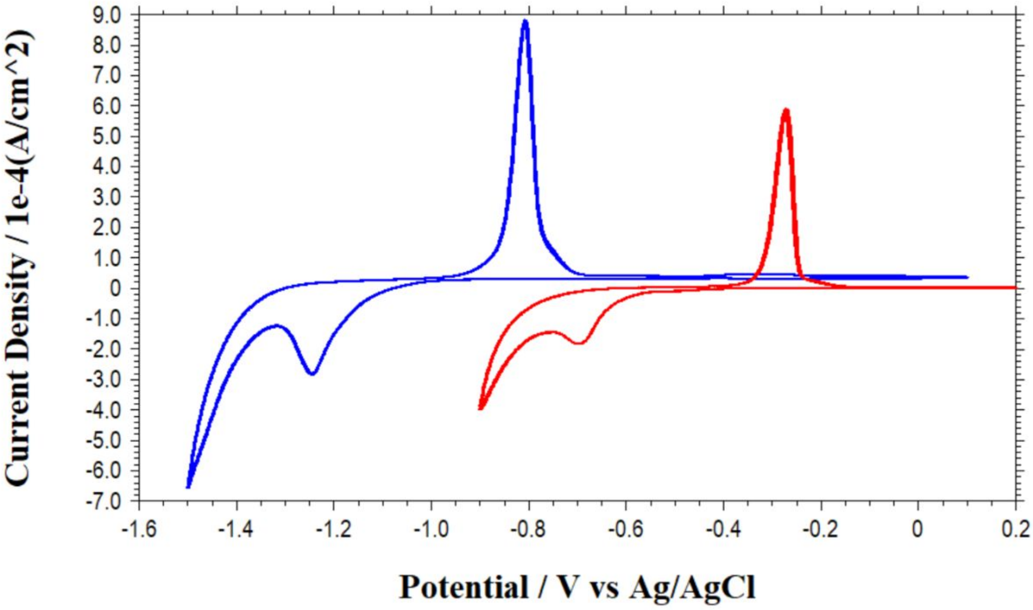

Figure S1. CVs recorded with an ordered Au(111) electrode in 0.1 M K_2SO_4 + 1 mM H_2SO_4 + 10 mM H_3BO_3 + 10 mM $NiSO_4$ using a Pt (blue line) and an Ag/AgCl (red line) reference electrode. These results show that the Pt potential scale is 550 mV more negative than that of the Ag/AgCl scale.

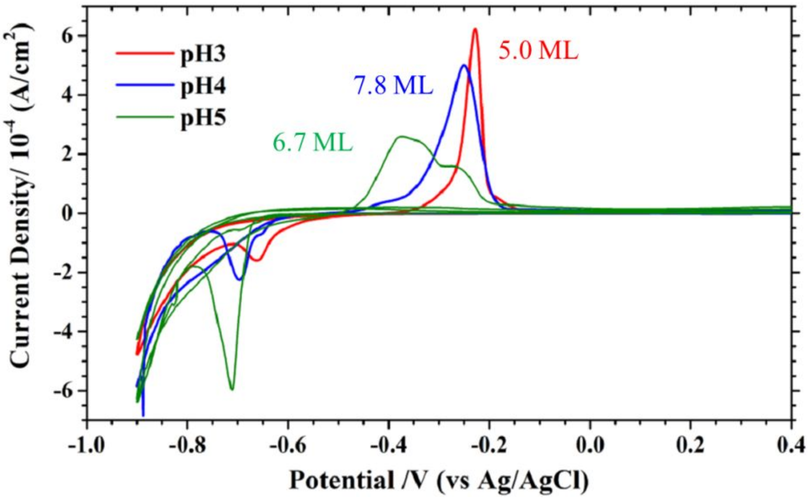

Figure S2. CVs recorded with an ordered Au(111) electrode in pH3 (red), 4 (blue), 5 (green) sulfate media (0.1 M K_2SO_4 + 10 mM $NiSO_4$ and + 10 mM H_3BO_3 with pH adjusted by adding 1 mM H_2SO_4). The Ni stripping peak shifts to more negative potentials and broadens with the increase of pH. The Ni deposit is the thickest in pH 4 sulfate.

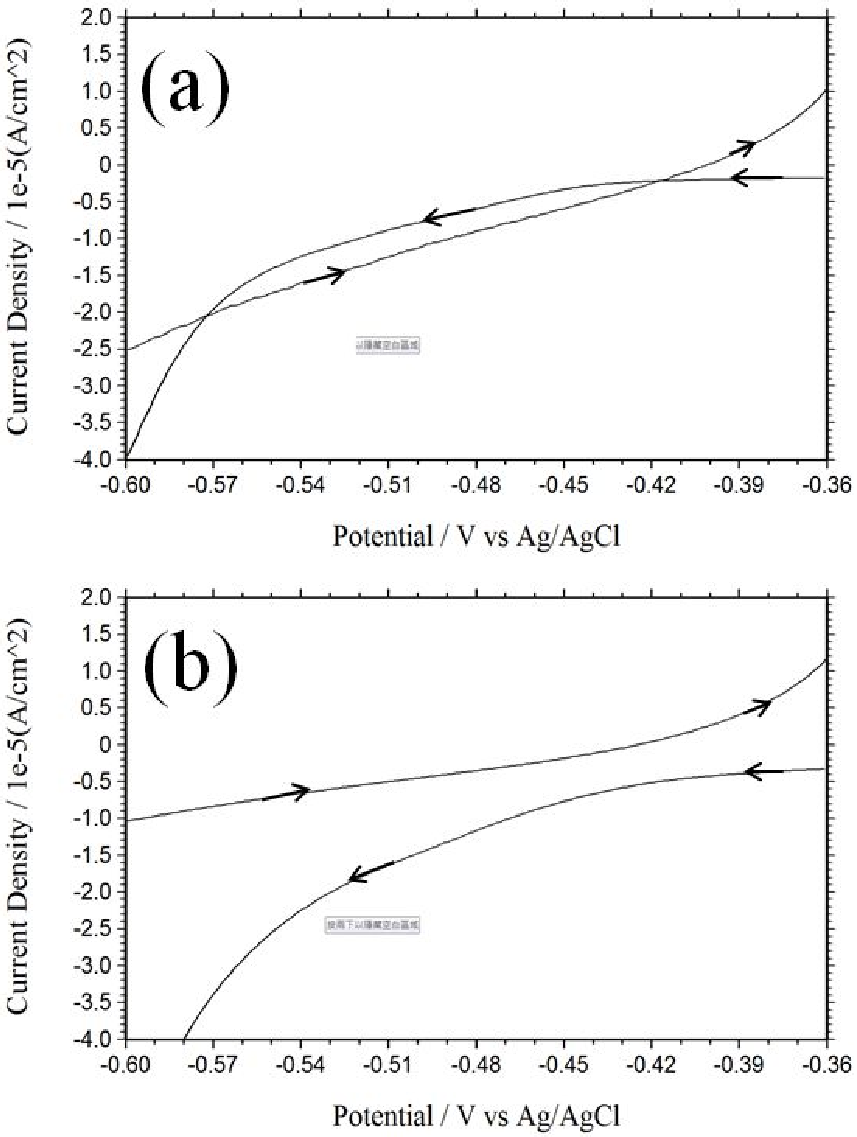

Figure S3. The first (a) and second (b) potential cycles recorded with Au(111) in 0.1 M K_2SO_4 + 1 mM H_2SO_4 + 10 mM H_3BO_3 + 10 mM $NiSO_4$, showing the hysteresis in the negative and positive scans. These CV profiles are presumed to reflect the changed surface structure of the Au(111) electrode, as indeed revealed by the STM images shown in Figure S3.

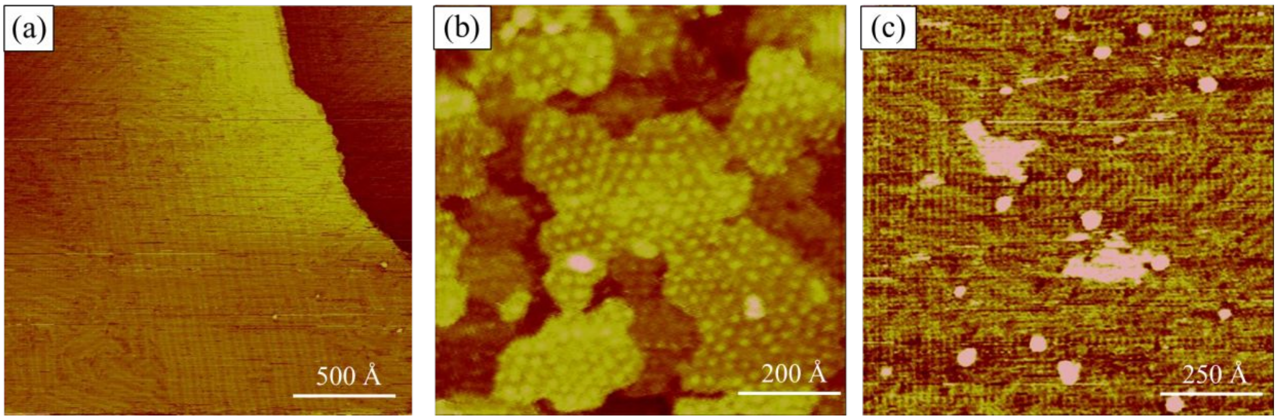

Figure S4. In situ STM images comparing the surface state of the Au(111) electrode in the first and second cycles of Ni deposition in 0.1 M K_2SO_4 + 1 mM H_2SO_4 + 10 mM H_3BO_3 + 10 mM $NiSO_4$. Panel (a) shows the pristine Au(111) electrode with the potential set at -0.55 V. The well – ordered reconstructed pattern (elongated lines) was decorated with some Ni nuclei imaged as tiny spots (a). Shifting the potential to -0.6 V caused Ni deposition in multilayer (b). The Ni deposit was largely removed by raising the potential to -0.3 V (c), which caused marked changes in the Au(111) surface. Short linear segments < 200 Å are obvious. These STM images were collected in the same experiment.

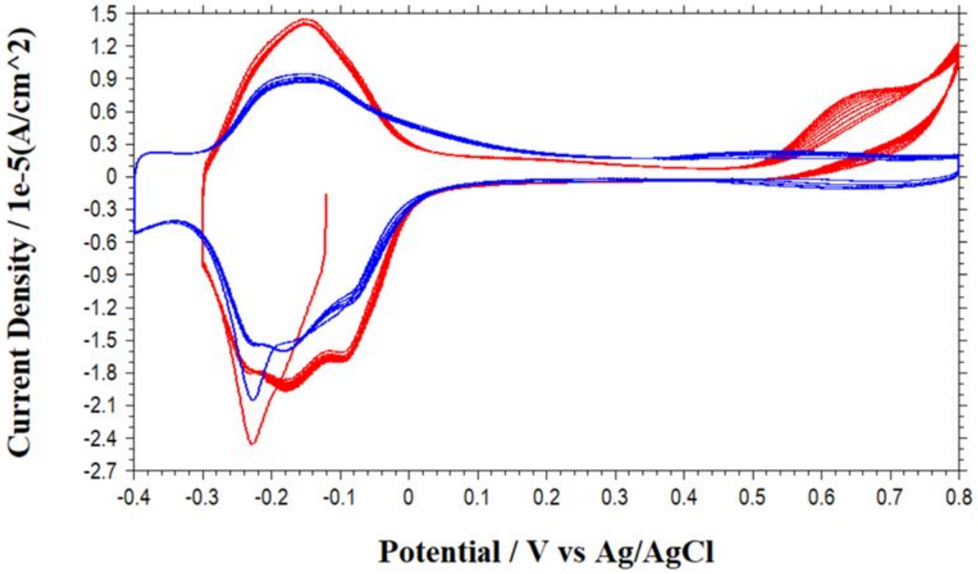

Figure S5. CVs recorded with Au(111) electrode in 0.1 M K_2SO_4 + 1 mM H_2SO_4 + 0.01 (blue) and 0.1 mM (red line) MMI, revealing the effect of [MMI] on the CV characteristics. Both CVs have broad peaks located between 0 and -0.3 V, which are associated with protonation and restructuring of the MMI adlayer. MMI molecule was irreversibly oxidized at potential > 0.5 V. The intensities of CV features are proportional to the amount of MMI molecule adsorbed on the electrode, reflecting dissimilar MMI's coverages on these samples.

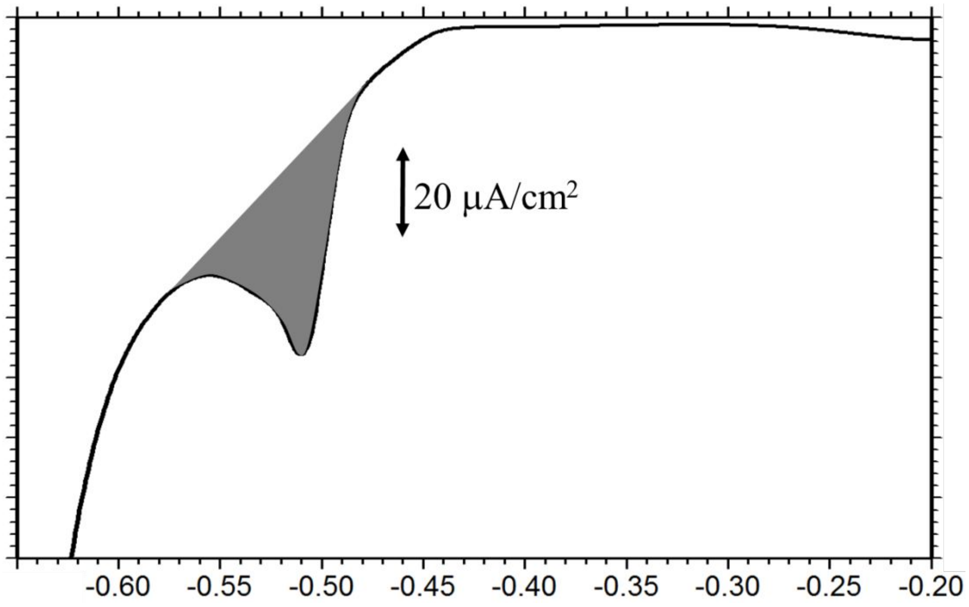

Figure S6. CVs recorded at 10 mV/s with Au(111) electrode pre-modified with 0.1 mM MMI in 0.1 M K_2SO_4 + 1 mM H_2SO_4 + 10 mM $NiSO_4$, featuring the pre-peak prior to the bulk deposition of Ni. This peak is presumed to the Ni UPD, which contains 130 μC/cm^2 charge.

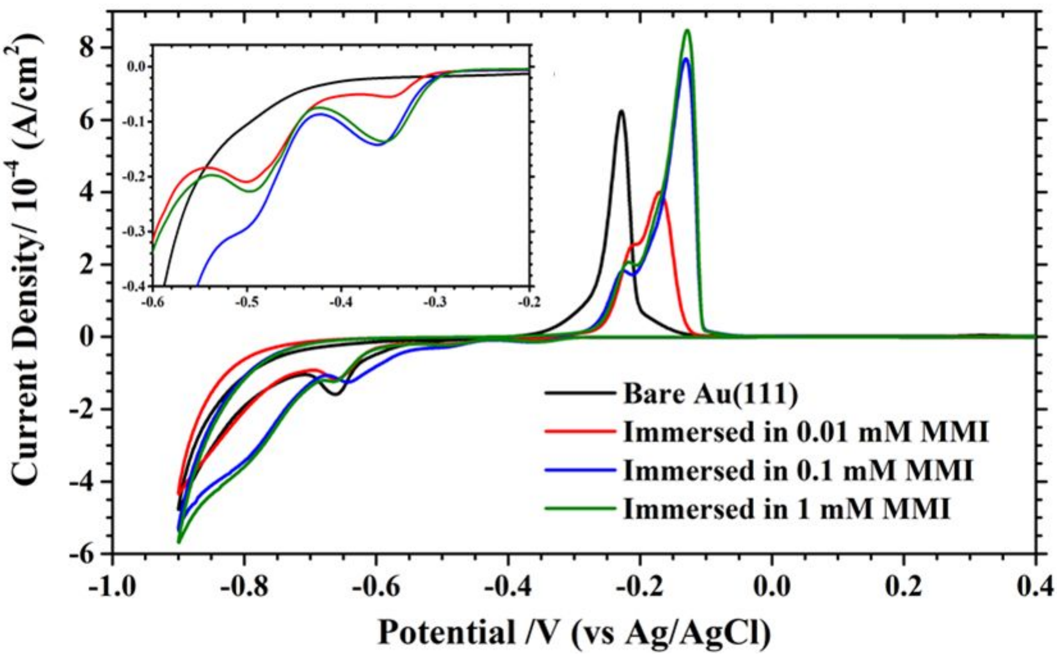

Figure S7. The 10th cycles recorded at 10 mV/s with Au(111) electrodes pre-modified with MMI in pH 3 sulfate solution + 10 mM $NiSO_4$. The amount of Ni deposit increased with potential cycles, reaching 4.9, 8.7, 9.4 MLs. The inset shows the first negative sweeps recorded with Au electrode modified with different [MMI].

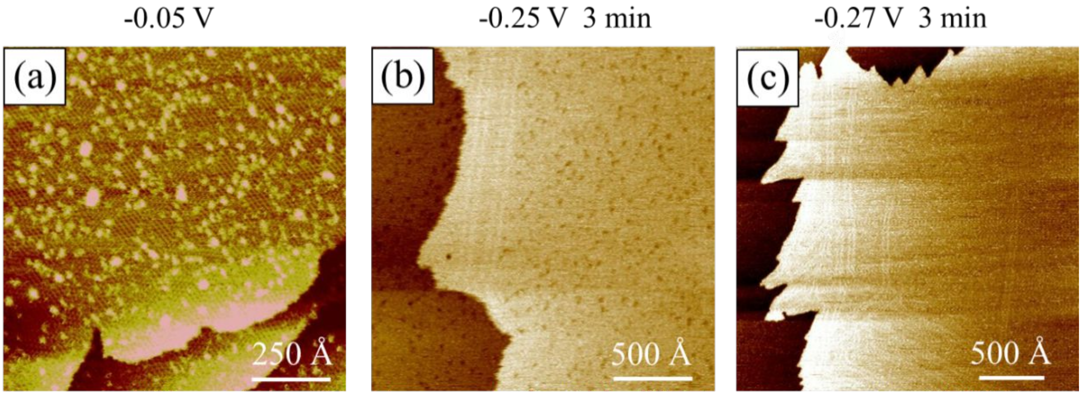

Figure S8. In situ STM images showing patchy ordered MMI structure on the Au(111) electrode at -0.05 V (a) in pH3 sulfate solution containing 10 μM MMI and 10 mM $NiSO_4$. MMI admolecules started to desorb from the Au(111) electrode at -0.25 V (b), revealing a pit – ridden Au surface. MMI desorption continued at -0.27 V to restore more the reconstructed Au(111) structure (c), but pitted (1 × 1) domains are still obvious.

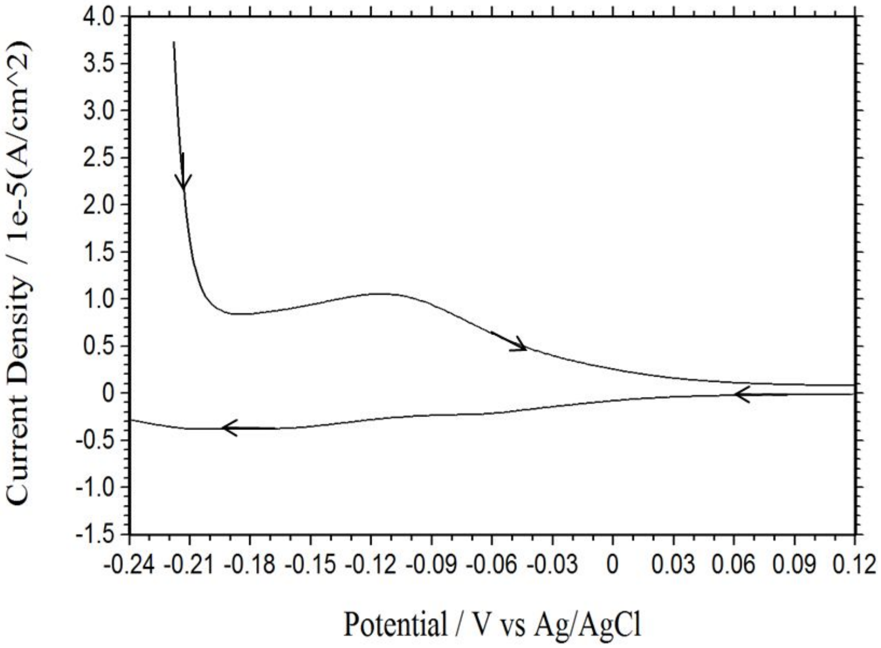

Figure S9. The stripping of Ni deposit from the Au(111) electrode in pH 3 sulfate + 10 mM $NiSO_4$ + 0.01 mM MMI. The broad peak seen at -0.12 V is attributed to stripping of the first Ni layer. Potential sweep rate = 10 mV/s.
